# Stimulus decay functions in action control

**DOI:** 10.1038/s41598-022-24499-6

**Published:** 2022-11-22

**Authors:** Christian Frings, Birte Moeller, Christian Beste, Alexander Münchau, Bernhard Pastötter

**Affiliations:** 1 Cogntive Psychology, Trier University, Universitätsring 15, 54296 Trier, Germany; 2 Cognitive Neurophysiology, Department of Child and Adolescent Psychiatry, Faculty of Medicine, TU Dresden, Fetscherstrasse 74, 01307 Dresden, Germany; 3 Institute of Systems Motor Science, University of Lübeck, Ratzeburger Allee 160, 23538 Lübeck, Germany

**Keywords:** Psychology, Human behaviour

## Abstract

When facing particular combinations of stimuli and responses, people create temporary event-files integrating the corresponding stimulus and response features. Subsequent repetition of one or more of these features retrieves the entire event-file, which impairs performance if not all features are repeated (partial-repetition costs). In the literature, different decay functions have been reported presumably dependent on the type of feature that is repeated (e.g. target vs. distractor features). Here, we use a variant of the S1R1-S2R2 and distractor-response binding task and analyze for the first time target-based and distractor-based event-file decay functions within the same task and sample. While we found evidence for decay functions and also stronger retrieval due to target than distractor repetitions, slopes of the decay functions were comparable suggesting that the decay process itself is equal irrespective of the type of stimulus feature that is repeated. Our study thereby confirms overarching approaches that summarize paradigm specific findings with the same set of core processes.

## Introduction

Humans organize perception and action in integrated representations of individual, distributed features. These integrated representations of sensorimotor episodes are compiled by binding features of actions and concurrent stimulation into *event-files*^1–6^. The entire set of features can be retrieved upon re-encountering one of the features, thus creating a cognitive short-cut for efficient action control as the retrieved response features might facilitate responding in the current situation.

In the laboratory, the influence of such event-file binding on action execution is usually measured with sequential tasks. At the first (the prime) event, stimulus- and response features are integrated, so that repetition of any of these features at the second (or probe) event can retrieve the previous episode thereby influencing responses to the probe. These so-called ‘binding effects’ occur via target stimuli and response integrations (e.g.^7^), but also for distractor stimuli (e.g.,^8,9^), effect stimuli (e.g.,^10^), and responses. Even tasks^11^, and control states^12^ can be integrated into event-files.

For the present purpose, it is important to note that depending on the type of binding effect, the duration for that the effect can be measured is variable. While response-irrelevant distractor stimuli are reliably integrated with responses (e.g.,^8,13,14^), distractor-response binding effects equally reliably decay within two seconds after integration^15–18^. Bindings between target stimuli and responses can still be measured four seconds after integration^19^, but seem to have fully decayed after about 5 s^20^. Neurophysiological evidence suggests that a decay of the neurophysiological correlates of an event file representation starts ~ 500 ms after an event file was built^21^. In contrast, bindings between individual responses do not seem to decay to a measurable extent within the first six seconds after integration^22^, also considering the neurophysiological level^21,23^, and data on control-state bindings seem to be in line with such response bindings^24^.

In this article we focus on *stimulus* binding effects. The differences in the stability between distractor-based and target-based binding effects is so far not well understood. There are arguments that attention might play a role here^25^, but one particular tacit assumption has never been challenged, namely that the decay function for all stimulus features is essentially the same. The recent *binding and retrieval in action control* (BRAC) framework argues to explain a plethora of paradigms with only few core processes^6^ and tacitly assumes that the decay function and the retrieval process is the same for all features. This assumption is what we want to test and challenge here.

To this end, we combine the S1R1-S2R2 task^7^ with the distractor-response binding task^8^ so that in the same task target-based and distractor-based binding effects can be measured. Attention allocation to target stimuli should further their integration and particularly the retrieval^26^ observable as a main effect for binding type (i.e. target-binding is expected to be way larger than distractor-binding). Varying the response-stimulus-interval (RSI) between prime and probe display should—according to previous studies^16,20^—lead to decreased binding effects for both types of effects (observable as a main effect for RSI). The crucial question is whether the decay is comparable (that is the interaction between RSI and binding type or the slope of the decay function). If the same decay function underlies distractor-based and target-based bindings we should not find an interaction. This would suggest that decay processes are independent of the type of stimulus features, which would lend support for overarching approaches that summarize paradigm specific findings with the same set of core processes—such as BRAC.

## Experiment

### Method

#### Participants

Distractor-response binding effects for horizontally flanking distractors ranged between *dz* = 0.7 and *dz* = 1.0 in past studies^27,28^. Binding effects of response and target feature are typically even larger than those between response and distractor feature (see^20^). Yet, effect sizes in decay rates in previous studies ranged between *dz* = 0.3 and *dz* = 1.2 so we planned for a sample size, sufficient to find at least a medium sized effect, *dz* = 0.4, assuming alpha = 0.05 and a power of 1 − ß = 0.90. A power analysis with the program G*Power revealed that at least 55 participants were necessary^29^. We recruited fifty-six students (46 women) from the University of Trier. Participants’ median age was 24 years with a range from 18 to 38 years. All participants took part in exchange for partial course credit or monetary compensation. The experiment followed the ethical guidelines of the University of Trier and was in accordance with the guidelines of the German Psychology Association (DGPs). We adhered the recommendations of the German Research Council (DFG) that waive the need for ethic approval of behavioral, non-invasive experiments with non-emotional stimuli. Informed consent was obtained. This study was not preregistered.

#### Design

The design comprised four within-subjects factors, namely response relation (response repetition vs. response change), target relation (repetition vs. change), distractor relation (repetition vs. change) and response-stimulus interval (RSI) between prime response R1 and presentation of probe stimulus S2 (500 ms vs. 2000 ms).

#### Materials

The experiment was conducted using the E-prime 2.0 software. Instructions were shown in white on black background on a standard TFT screen. Target stimuli were the letters D and K, presented in red, distractors were the letters G and H, presented in green. R1 response identity (left or right) was indicated via three left or right pointing arrows (<<<or>>>). All letters subtended a horizontal visual angle of 0.6° to 0.7° and a vertical visual angle of 0.6°. Each array of left or right pointing arrows had a horizontal visual angle of 1.7° and a vertical visual angle of 0.5°. Viewing distance was approximately 60 cm.

#### Procedure

Participants were tested individually in soundproof chambers. Instructions were given on the screen. Participants were instructed to place their left index finger on the D key and their right index finger on the K key of a standard computer keyboard. Participants always saw the letter D or the letter K that was horizontally flanked by two additional letters (either G on both sides or H on both sides). These letter-triplets were presented in the center of the display. The procedure was adapted from^7^. Participants’ task was to execute a pre-cued response R1 upon presentation of S1 and to respond to the identity of the central target letter by pressing a key with the corresponding finger upon presentation of S2. Participants were instructed to react as quickly and as correctly as possible.

**Figure 1:**
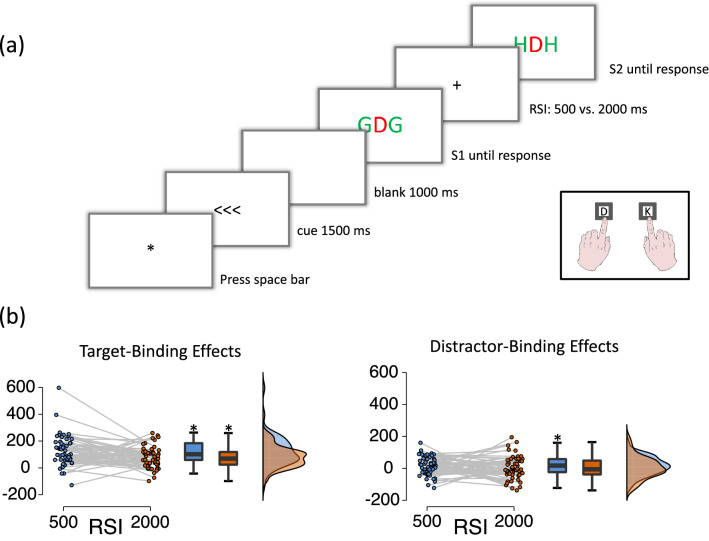
(**a**) Sequence of events in one trial. Participants executed a pre-cued and prepared response upon presentation of S1 and gave a discrimination response to S2 (the central letter). Flanking distractor letters were to be ignored. This is an example for a response repetition trial with distractor change. White is depicted in black and black is depicted in white. Stimuli are not drawn to scale. (**b**) Target-based binding effects and distractor-based binding effects in milliseconds as a function of RSI (blue indicates RSI 500 ms while orange indicates RSI 2000 ms) depicted at the level of individual effects, at the group level via boxplots, and as a frequency distribution. Binding effects are computed as the interaction of stimulus relation × response relation. * indicates *p*-values < 0.05.

Each trial was started self-paced by the participant and featured a S1R1-S2R2 sequence with the following events (see Fig. 1a). After participants started the trial by pressing the space bar, three arrows (<<<or>>>) appeared for 1500 ms in the middle of the screen, indicating the response required upon S1 presentation. After a 1000 ms blank interval, the S1 letter triplet appeared and stayed on the screen until the participant responded. Then a fixation cross was presented for either 500 ms or 2000 ms (according to the RSI condition), before the S2 letter-triplet appeared and stayed on the screen until the participant responded to the target identity. In response repetition trials, the same response was indicated by the cue (R1) and required by the identity of the target letter in S2 (R2); in response change trials, R1 and R2 differed. In target repetition trials, the S2 target was presented as the central letter in S1; in target change trials the S2 target differed from the central letter in S1. Note that because R1 was cued while R2 was a discrimination response, it is possible to separately repeat or change targets and responses. In distractor repetition trials the flanking letters were repeated from S1 to S2 and in distractor change trials the flanking letters differed between S1 and S2. The orthogonal variation of the factors response relation, target relation and distractor relation resulted in eight conditions: response repetition with target repetition and distractor repetition (RrTrDr), response repetition with target repetition and distractor change (RrTrDc), response repetition with target change and distractor repetition (RrTcDr), response repetition with target change and distractor change (RrTcDc), response change with target repetition and distractor repetition (RcTrDr), response change with target repetition and distractor change (RcTrDc), response change with target change and distractor repetition (RcTcDr), and response change with target change and distractor change (RcTcDc). Each participant completed one experimental block including a 500 ms RSI (128 trials) and another including a 2000 ms RSI (128 trials) between R1 execution and S2 presentation onset. Block order was counterbalanced across participants. Before the experiment started, participants worked through 32 practice trials with the first block’s RSI. Before the second block started, participants worked through an additional 8 practice trials that included the RSI of the second block. Practice trials were a subset of the experimental trials.

### Results

Individual reaction time distributions were smoothed by applying non-parametric outlier detection to the lower and upper part of the distribution (according to^30^). In addition, only trials with correct prime and probe responses were considered for RTs. These criteria led to the exclusion of 13.1% (SD 12.6%) of all trials.

RTs and error rates were combined into inverse efficiency scores (see Supplement for RT and error data) as probe error rate was only 3.6% (SD 2.1%)^31^. Target-binding effects were built by computing the interaction of response relation × target relation, distractor-binding effects were built by computing the interaction of response relation × distractor relation. Target-based binding effects were significant for both RSIs, *t*(54) = 8.12, *p* < 0.001, for the RSI 500 ms, and *t*(54) = 7.411, *p* < 0.001, for the RSI 2000 ms interval while distractor-based binding effects were significant for the RSI 500 ms condition, *t*(55) = 2.18, *p* = 0.033, but not for the RSI 2000 ms condition, *t*(55) = 0.33, *p* = 0.741. These binding effects were then submitted to a 2 (stimulus type: target vs. distractor) × 2 (RSI: 500 vs. 2000 ms) repeated measures ANOVA. The main effect for stimulus type was significant, *F*(1,54) = 65.613, *p* < 0.001, ω^2^ = 0.359, showing that binding effects were larger for targets than for distractors (this can be seen when you compare the average target binding effect with the average distractor binding effect as reported for the t-tests above). The main effects of RSI was also significant, *F*(1,54) = 8.132, *p* = 0.006, ω^2^ = 0.054, showing that binding effects were larger for shorter RSIs than for longer RSIs, thus replicating previous findings on decay functions (this can be seen when you compare the binding effects at short RSI with the ones at long RSI as reported for the t-tests above). RSI and stimulus type did not interact, *F*(1,54) = 2.435, *p* = 0.124, ω^2^ = 0.012 (see Fig. 1b). To corroborate these findings a Bayesian repeated measures ANOVA was run. A model that included only both main effects was the best model (BF_M_ = 4.88), while adding the interaction term did not differ from the best model (BF_10_ = 0.661; according to BF conventions^32^). This pattern reflects that the differences between target binding effects at short and long RSIs is comparable to the differences between distractor binding effects at short and long RSIs.

## Discussion

As expected we observed and replicated that targets produce larger binding effects than distractors (^25^, e.g.,^26^) and that binding effects on average diminish over time^16,20^. More intriguing is that we did not find an interaction of stimulus type and RSI suggesting that the decay function for stimulus features is essentially the same for targets and distractors that means the binding effects for targets and distractors diminish to a comparable degree over time albeit they start at different levels. Put differently, the tacit assumption of only one decay function for stimulus features that is inherent in most action control approaches was confirmed. The observed differences for distractor-based and target-based binding effects can be attributed to differences in attention allocation—obviously relevant stimuli receive more attention and hence might have just a head-start for the binding and retrieval while the decay function itself is independent of the stimulus type.

Yet, we have to acknowledge that a study with more test power might find differences in the decay function. The crucial test for the different decay functions is the Bayesian analysis and this analysis suggested that the model without the interaction term is the best while adding the interaction term does not improve the model fit—still the evidence for this null effect was rather small or anecdotal according to Schönbrodt and Wagenmarkers^33^. Further research with more test power and more time points might pinpoint possible differences in stimulus dependent decay functions. However, our study suggests that if such differences exist they are small.

The different decay functions for stimulus-based versus response-based bindings as noted in the introduction might be due to the fact that these bindings serve different functions. Following research on hierarchical action representations (see^34,35^), response-response bindings might enable the formation of complex action representations, which are supposed to group sub-elements of an extended action by chunking multiple simple responses. For such higher-level representations, temporal stability is relevant, because these representations merge temporally distant events. However, at the level of stimulus representations, quick disintegration of stimulus–response bindings should prevent interference between individual episodes (see^20^).

The tacit assumption of only one decay function for stimulus features in action control as suggested by the BRAC framework^6^ or the *theory of event-coding* (TEC; 1) was confirmed. This is an important theoretical advance as overarching approaches trying to summarize and integrate action control paradigms need evidence for core processes that are not paradigm- or stimulus-specific. Stimuli are stimuli—differences between them are due to attentional allocation or their role as response-relevant or response-irrelevant stimuli etc. but the underlying decay and retrieval function is the same (Supplementary Tables).

## Supplementary Information

Supplementary Tables.

## Data Availability

Raw data and aggregated data are available on OSF, Link: https://doi.org/10.17605/OSF.IO/Z6HQ8.
