# Supplementary material for: Stimulus decay functions in action control
Source: Sci Rep. 2022 Nov 22;12:20139. doi: 10.1038/s41598-022-24499-6 (PMC9684409; doi:10.1038/s41598-022-24499-6)
Supplement: Supplementary file 1 — Supplementary Tables. [file 41598_2022_24499_MOESM1_ESM.docx]

**Table S1. Mean binding effects with SD in parenthesis for reaction times (in milliseconds) and error rates (in %) as a function of stimulus type x RSI.**

|  |  | Reaction times | |  |  | | Error rates | |
| --- | --- | --- | --- | --- | --- | --- | --- | --- |
|  | *RSI 500* |  | *RSI 2000* |  | *RSI 500* |  | | *RSI 2000* |
| Target-based | 60 (58) |  | 49 (53) |  | 9 (11) |  | | 4 (7) |
| Distractor-based | 3 (33) |  | 1 (41) |  | 2 (8) |  | | 1 (8) |

**Table S2. Repeated measures 2 (stimulus type) x 2 (RSI) ANOVAs for binding effects in reaction times and error rates.**

|  | *Reaction times* |  | *Error rates* |
| --- | --- | --- | --- |
| Main effect stimulus type | *p* < .001, ω^2^ = 0.364 |  | *p* < .001, ω^2^ = 0.155 |
| Main effect RSI | *p* = .282, ω^2^ = 0.002 |  | *p* = .002, ω^2^ = 0.077 |
| Interaction | *p* = .416, ω^2^ <. 001 |  | *p* = .177, ω^2^ = 0.010 |
